# Study of the Aminoglycoside Subsistence Phenotype of Bacteria Residing in the Gut of Humans and Zoo Animals

**DOI:** 10.3389/fmicb.2015.01550

**Published:** 2016-01-11

**Authors:** Teresita de J. Bello González, Tina Zuidema, Gerrit Bor, Hauke Smidt, Mark W. J. van Passel

**Affiliations:** ^1^Laboratory of Microbiology, Wageningen UniversityWageningen, Netherlands; ^2^RIKILT, Wageningen UniversityWageningen, Netherlands; ^3^National Institute of Public Health and EnvironmentBilthoven, Netherlands

**Keywords:** antibiotic resistance, antibiotic subsistence, antibiotic subsistence phenotype, aminoglycosides, single carbon source

## Abstract

Recent studies indicate that next to antibiotic resistance, bacteria are able to subsist on antibiotics as a carbon source. Here we evaluated the potential of gut bacteria from healthy human volunteers and zoo animals to subsist on antibiotics. Nine gut isolates of *Escherichia coli* and *Cellulosimicrobium* sp. displayed increases in colony forming units (CFU) during incubations in minimal medium with only antibiotics added, i.e., the antibiotic subsistence phenotype. Furthermore, laboratory strains of *E. coli* and *Pseudomonas putida* equipped with the aminoglycoside 3′ phosphotransferase II gene also displayed the subsistence phenotype on aminoglycosides. In order to address which endogenous genes could be involved in these subsistence phenotypes, the broad-range glycosyl-hydrolase inhibiting iminosugar deoxynojirimycin (DNJ) was used. Addition of DNJ to minimal medium containing glucose showed initial growth retardation of resistant *E. coli*, which was rapidly recovered to normal growth. In contrast, addition of DNJ to minimal medium containing kanamycin arrested resistant *E. coli* growth, suggesting that glycosyl-hydrolases were involved in the subsistence phenotype. However, antibiotic degradation experiments showed no reduction in kanamycin, even though the number of CFUs increased. Although antibiotic subsistence phenotypes are readily observed in bacterial species, and are even found in susceptible laboratory strains carrying standard resistance genes, we conclude there is a discrepancy between the observed antibiotic subsistence phenotype and actual antibiotic degradation. Based on these results we can hypothesize that aminoglycoside modifying enzymes might first inactivate the antibiotic (i.e., by acetylation of amino groups, modification of hydroxyl groups by adenylation and phosphorylation respectively), before the subsequent action of catabolic enzymes. Even though we do not dispute that antibiotics could be used as a single carbon source, our observations show that antibiotic subsistence should be carefully examined with precise degradation studies, and that its mechanistic basis remains inconclusive.

## Introduction

Antibiotic resistance is a global health problem, and resistance is prevalent in bacteria isolated from both human and animal sources (van den Bogaard and Stobberingh, 2000; Sommer et al., 2009). Also, other natural habitats, for example soil, represent a common reservoir of resistance genes (Dantas et al., 2008). Recent metatranscriptome analyses have revealed that antibiotic resistance genes are expressed in a broad range of natural habitats, even in the absence of obvious antibiotic selection pressure (Versluis et al., 2015). Furthermore, metagenomic studies of ancient environments have revealed that antibiotic resistance is a natural phenomenon that predates the anthropogenic selective pressure of clinical antibiotic use (D’Costa et al., 2011).

It has long been speculated that, for example in clinically relevant strains, genes conferring resistance to aminoglycoside antibiotics were derived from organisms producing aminoglycosides, suggesting that members of the *Actinomycetes* could have provided the initial pool of aminoglycoside resistance genes (Benveniste and Davies, 1973; Wright, 2007). Aminoglycosides are useful in the treatment of Gram-negative aerobic bacilli, staphylococci, and other Gram-positive bacterial infections (Yao and Moellering, 2007). The initial site of aminoglycoside action is the outer bacterial membrane, where the cationic antibiotic molecules create fissures in the outer cell membrane. These fissures result in leakage of intracellular contents, and enhanced antibiotic uptake. Once inside the bacterial cell, aminoglycosides inhibit protein synthesis by binding to the 30S ribosomal subunit (Gonzalez et al., 1998). Resistance to aminiglycosides is often due to enzymatic inactivation by acetyltransferases, nucleotidyltransferases and phosphotransferases. Other resistance mechanisms include loss of permeability, structural alteration of the ribosomal target and the presence of efflux pumps (Azucena and Mobashery, 2001). Streptomycin, a representative of aminoglycoside antibiotics produced naturally by bacteria, has been shown to participate in microbial survival pathways. These pathways can be defined as the capacity of bacterial metabolism to modulate antibiotic resistance (Martinez and Rojo, 2011). This could indicate that aminoglycosides, apart from inhibiting bacterial growth, could stimulate the acquisition of aminoglycoside resistance genes. This can play an important role in the survival of microorganisms, as indicated for the acetyltransferase involved in aminoglycoside resistance in *Providencia stuartii* (Goldberg et al., 1999; Barlow and Hall, 2002).

Recently a large and diverse group of bacteria from soil, seawater, and the gut of humans and farm animals were found to not merely resist the toxic effects of antibiotics, but also to use antibiotics including aminoglycosides as a single carbon source. This phenotype is commonly referred to as “antibiotic subsistence” (Dopazo et al., 1988; Dantas et al., 2008; Barnhill et al., 2011; Xin et al., 2012). In addition, the concept of bacteria subsisting on antibiotics has been referred to as “antibiotic-resistant extremophiles” (Gabani et al., 2012) or “antibiotrophs” (Woappi et al., 2014) These alternative terms depict the microorganisms as being able to subsist under harsh environmental conditions, e.g., elevated antibiotic concentrations or the use of antibiotics as the sole carbon source. In disagreement with the accumulating body of literature supporting the possibility of bacterial subsistence on antibiotics, Walsh et al. (2013) tested whether soil bacteria could subsist on antibiotics. As no degradation of antibiotics occurred, Walsh et al. (2013) concluded that soil bacteria could not utilize antibiotics (including streptomycin, trimethoprim, penicillin, and carbapenicillin) as a carbon source.

To date, no genes have been identified that could enable bacteria to use antibiotics as a single carbon source, and therefore the relationship between antibiotic resistance and antibiotic subsistence remains unclear (Dantas and Sommer, 2012). To this end, and since the gut microbiota of humans and animals has been described as a reservoir of antibiotic resistance, we studied the potential of gut bacteria to display the antibiotic subsistence phenotype using a range of antibiotics. Almost all of the bacteria able to subsist on antibiotics grew on an aminoglycoside, and therefore we focused on aminoglycosides to address mechanistic aspects of the subsistence phenotype that could be readily approached using laboratory model organisms.

## Materials and Methods

### Samples and Antibiotics Used

We evaluated the antibiotic subsistence phenotype of bacteria subsisting on a range of antibiotics: ampicillin, chloramphenicol, erythromycin, kanamycin, streptomycin and tetracycline (1mg/ml) (Sigma-Aldrich, Zwijndrecht, The Netherlands). Fecal samples from two healthy human volunteers and six species of exotic zoo animals (Burgers’ Zoo – Arnhem, the Netherlands) with no previous antibiotic administration (6 months) were used as inocula (**Table 1**). Fecal samples from zoo animals were taken by the zookeepers following internal standard regulations. The samples were collected immediately after defecation into a sterile container, and then stored at 4°C (for 0.5–4 h) before being transferred to –80°C.

**Table 1:** Human and zoo animal fecal isolates with subsistence phenotype on antibiotics.

Isolate (% 16S rRNA gene identity)	Source (Latin name)	Resistant to^∗^	Subsisting on^∗^	Accession number
*Escherichia coli* (100)	Human 1 *(Homo sapiens)*	AMP, TET, E, KAN, STR	STR	KT989026
*E. coli* (100)	Human 2 *(Homo sapiens)*	AMP, TET, KAN, STR, CL	KAN	KT989027
*Cellulosimicrobium* sp. (99)	Chimpanzee *(Pan troglodytes)*	AMP, TET KAN, STR	KAN	KT989030
*Cellulosimicrobium* sp. (100)	Chimpanzee *(Pan troglodytes)*	AMP, TET KAN, STR	STR	KT989029
*E. coli* (100)	Baringo giraffe *(Giraffe camelopardalis rothschildi)*	TET, KAN, STR	KAN	KT989033
*E. coli* (100)	Asian elephant (*Elephas maximus*)	AMP, TET, E, KAN, STR	KAN	KT989034
*Escherichia coli* (100)	Malayan sun bear*(Ursus malayanus)*	KAN, STR	KAN	KT989035
*E. coli* (100)	Sumatran tiger *(Panthera tigris sumatrae)*	AMP, TET, E, KAN, STR	KAN, E	KT989032
*Cellulosimicrobium* sp. (99)	Warthog *(Phacochoerus africanus)*	AMP, KAN, STR	KAN	KT989028


Agent abbreviation*: AMP, Ampicillin; TET, Tetracycline; E, Erythromycin; KAN, Kanamycin; STR, Streptomycin; and CL, Chloramphenicol.

### Isolating Bacteria with the Subsistence Phenotype

Fecal samples (∼200 mg) were suspended in 5 ml of M9 minimal salts medium (Sigma–Aldrich) and centrifuged twice (5 min at 18,400 *g*) to prevent carry-over of dissolved carbon from the fecal material. Washed bacterial cells were then suspended in 5 ml of fresh M9 medium, and 50 μl inoculated into 5 ml M9 medium supplemented with 1 mg/ml of a single antibiotic (98–99% purity) and incubated at 37°C for 24 h. Then, the cultures were serially transferred twice to a fresh media with antibiotic, followed by plating on Luria Broth agar (LB agar), to quantify the bacterial growth based on enumeration of colony forming units (CFU) on the LB plates were counted after 8, 24, 48 h of incubation at 37°C. The subsistence phenotype criteria was identified based on a twofold increase of CFUs over multiple transfers. A single colony was selected and tested to confirm the subsistence phenotype. Glucose (1 mg/ml) was used as a positive control, while M9 medium lacking any carbon source served as negative control for growth. All experiments were performed in duplicate.

### Identification of Bacterial Isolates with the Subsistence Phenotype

Bacteria subsisting on antibiotics were selected for DNA amplification using the 27F and 1492R primers. PCR was carried out with FastStart Taq DNA polymerase (Roche) in a reaction mixture containing 10X Fast Taq buffer + MgCl_2_, dNTPs (10 mM each, Roche), 10 pmol of both primers in a final volume of 49 μl; finally add the template of DNA (1 μl). For the amplification reaction, after 5 min at 95°C, 35 identical cycles (30 s of denaturation at 95°C, 40 s of annealing at 52°C, 90 s of elongation at 72°C) were followed by a final elongation step of 7 min at 72°C. The amplified fragments were selected for partial sequence analysis of the 16S rRNA gene (∼800 bp) using the 1392R primer, and sequences were deposited in GenBank with accession numbers KT989026, KT989027, KT989028, KT989029, KT989030, KT989031, KT989032, KT989033, KT989034, KT989035 (**Table 1**). Furthermore, all isolates were tested for their antibiotic resistance phenotype by dilution agar test as recommended by Clinical and Laboratory Standards Institute (2014).

### Experimental Controls to Differentiate Between Aminoglycoside Resistance and the Subsistence Phenotype

In order to differentiate between antibiotic resistance and antibiotic subsistence, we used transformants containing a gene encoding aminoglycoside 3′ phosphotransferase II (APH (3′) II) (Berg et al., 1975), one of the most common aminoglycoside-modifying enzymes in prokaryotes, as a control. In detail, chemically competent cells of two different strains of *E. coli* (DH5α and TOP10) were transformed by heat-shock with cloning vectors pRSF-1b (Novagen, Billerica, MA, USA) and pCR-2.1TOPO (Invitrogen, Carlsbad, CA, USA), respectively, both containing an APH (3′) II gene. Also, we used *Pseudomonas putida* TEC1 transformed with the cloning vector pUTmini-Tn5-Km1 (de Lorenzo et al., 1990; Leprince et al., 2012). Transformed and non-transformed strains were tested for their ability to resist and subsist on the aminoglycoside antibiotics kanamycin and neomycin using the protocol described above.

### Effect of Deoxynojirimycin (DNJ) on the Aminoglycoside Subsistence Phenotype

To evaluate the involvement of glycosyl hydrolases (GH) in the subsistence phenotype on aminoglycoside we selected deoxynojirimycin (DNJ) (Laboratory of Organic Chemistry, Leiden University, The Netherlands), which is one of the simplest natural carbohydrate mimics that can competitively inhibit specific glycosidic enzymes (Hughes and Rudge, 1994). We tested the capacity of *E. coli* (DH5α) transformed with pRSF-1b plasmid-encoded APH (3′) II gene, to grow on kanamycin or glucose (1 mg/ml) as a single carbon source in the presence of DNJ (range of 0.00001–10 mM of DNJ) and monitored growth for 24 h. All the experiments were performed in triplicate and used 96-well plates. Growth was measured by OD = 600 nm for 24 h continuously during incubation at 37°C with agitation at 75 rpm.

### Kanamycin Degradation by *Escherichia coli*

To investigate kanamycin degradation by *E. coli* we performed an LC-MS/MS analysis. The experimental control was carried out using *E. coli* (DH5α) with and without cloning vector pRSF-1b in the presence of kanamycin (99.25% Kanamycin A Sulfate, EvoPure^TM^, GENTAUR Netherlands) (1 mg/ml). An aliquot was taken at 0, 4, 8, 24 h, and analyzed in duplicate using LC-MS/MS. In detail, the samples were diluted 100 times in 0,065% heptafluorbutyric acid, with an expected concentration of 10 mg/L. Octamethylkanamycine was added as an internal standard to the diluted samples at a concentration of 10 mg/L. Fifty microliter of the diluted sample was injected using a 2690 separations module high-performance liquid chromatography (HPLC) system (Waters Corporation, USA) coupled to a Quattro Micro tandem mass detector (Waters-Micromass, Manchester, UK). For the analysis samples were separated using a Symmetry C18 (150 mm × 3 mm, 5 μm) chromatographic column from Waters (Milford, PA, USA) working at 30°C and at a flow rate of 0.4 ml/min. The mobile phase was water containing 0.065% heptafluorbutyric acid (A) mixed on a gradient mode with methanol containing 0.065% heptafluorbutyric acid (B), as follows: initiated at 100% A, from 100 to 55% A in 5 min, from 55 to 40% A in 11.5 min, kept isocratic at 60% B for 5 min, from 60% B to 0% B in 1 min for equilibration of the column (initial conditions). The mass spectrometer was operated in electrospray positive mode, and data acquisition was in multiple reactions monitoring mode (MRM). Source settings were as follows: capillary voltage 2.7 kV, cone voltage 25 V, source temperature 120°C, desolvation temperature 400°C, cone nitrogen gas flow 60 L/h, desolvation gas flow 600 L/h. Argon was used as the collision gas at 3.2 × 10^-3^ mbar. Calibration was done by means of a calibration curve (0, 2, 5, 10, and 20 mg/L) in 0.065% heptafluorbutyric acid. Quantification of kanamycin in the samples was done on the calibrators by means of isotope dilution using octamethylkanamycin. The bacterial culture was also plated on LB agar for growth assessment (CFU/ml) as described above.

## Results

### Gut Bacteria of Human and Zoo Animals Displayed Subsistence Phenotype

Nine isolates from human and animal fecal samples displayed subsistence phenotypes when cultivated with a single antibiotic as the sole carbon source: six on kanamycin, two on streptomycin, and one isolate displayed the subsistence phenotype on both erythromycin and kanamycin (**Table 1**). The subsistence phenotype was measured by plating and counting CFU increases, with a twofold increase of CFUs used to identify the phenotype. The isolates were classified by partial sequence analysis of 16S rRNA genes, and seven isolates were identified as *E. coli* and three as *Cellulosimicrobium* sp. The *Cellulosimicrobium* sp. are members of the family *Promicromonosporaceae* within the *Actinobacteria*, and were most closely related to *Cellulosimicrobium cellulans* and *C. funkei* (**Table 1**), which are all related to human pathogens (Funke et al., 1995; Kaper et al., 2004; Petkar et al., 2011). All nine isolates were resistant to two or more of the following antibiotics: ampicillin, chloramphenicol, tetracycline, erythromycin, streptomycin, and kanamycin (**Table 1**).

### Experimental Controls to Differentiate Aminoglycoside Resistance and Subsistence Phenotype

Since nine isolates displayed the subsistence phenotype on aminoglycosides, mainly kanamycin, we included an experimental control in an attempt to differentiate between antibiotic resistance and antibiotic subsistence. This was performed by equipping laboratory strains with a plasmid-encoded APH (3′) II gene. All transformants of *E. coli* and *P. putida*, but none of the non-transformed strains, displayed the subsistence phenotype on kanamycin and neomycin (**Table 2**). Growth of the strains on glucose was similar to that in the presence of aminoglycosides, whereas no growth was observed in M9 medium to which no carbon source was added (**Table 2**).

**Table 2:** Growth experiments (48 h, performed in duplicate) of non-resistant and resistant *E. coli* and *P. putida* strains on media containing no carbon source, glucose, or aminoglycosides (kanamycin, neomycin) in M9 minimal salts medium.

	M9	M9 + Glucose 1 mg/ml	M9 + Kanamycin 1 mg/ml	M9 + Neomycin 1 mg/ml
***Escherichia coli***
DH5α	–	+	–	–
DH5α + pRSF-1b	–	+	+	+
DH5α + pCR-2.1 TOPO	–	+	+	+
TOP10	–	+	–	–
TOP10 + pRSF-1b	–	+	+	+
TOP10 + pCR-2.1 TOPO	–	+	+	+
***Pseudomonas putida***
TEC1	–	+	–	–
TEC1 + pUTmini-Tn5-Km1	–	+	+	+


*Gray highlighted boxes indicate strains showing subsisting phenotype on aminoglycosides. The + indicates growth, – indicates no growth.*

### Effect of Deoxynojirimycin on the Aminoglycoside Subsistence Phenotype

**FIGURE 1:**
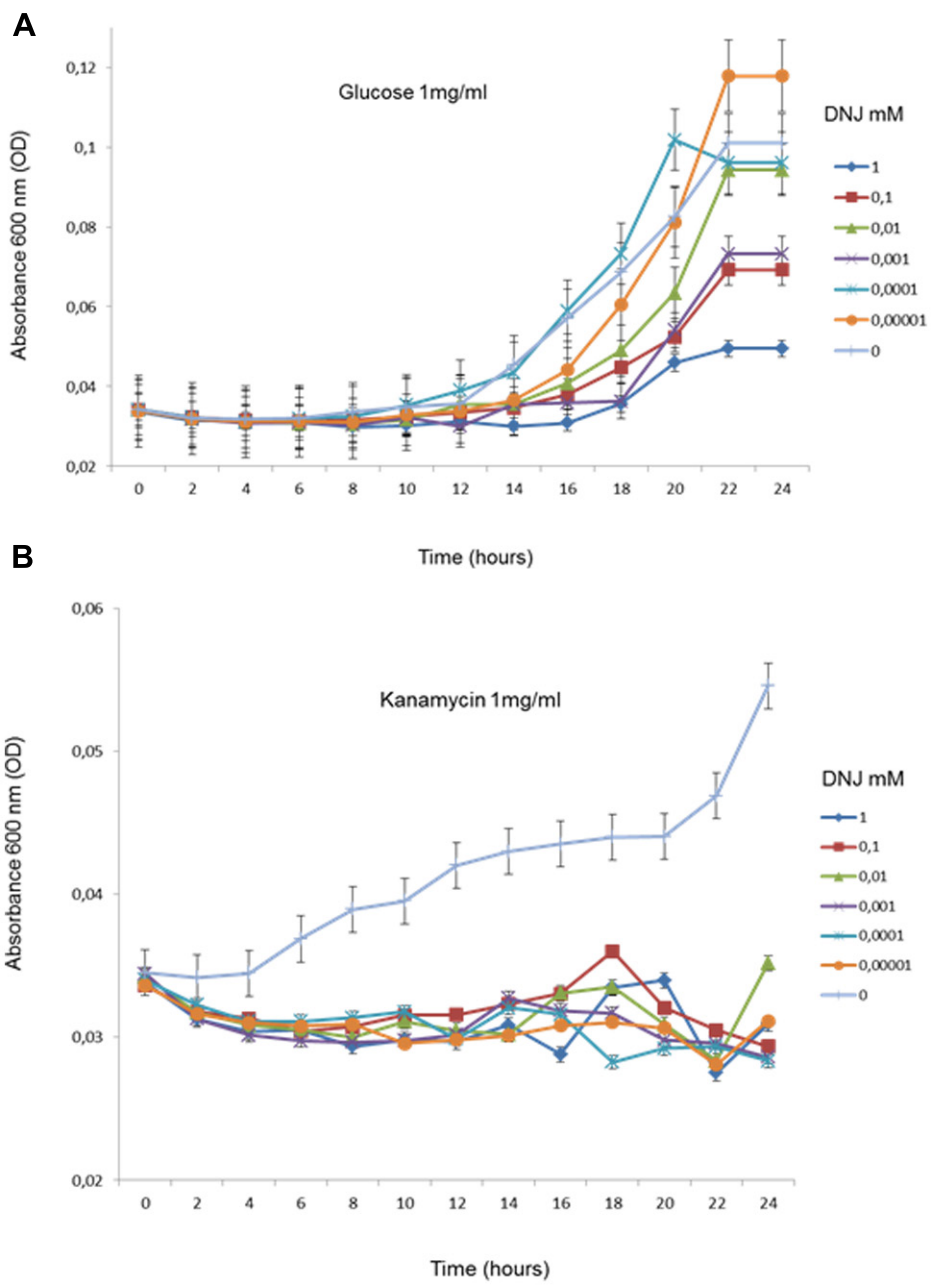
Growth dynamics (in triplicate) of transformed *E. coli* in M9 medium containing glucose (1 mg/ml) (A) and kanamycin (B) in the presence of different concentrations of DNJ (in mM).

In order to evaluate the involvement of GH in the subsistence phenotype on aminoglycoside, we tested the capacity of *E. coli* (DH5α) transformed with pRSF-1b plasmid- encoded APH (3′) II gene to grow on kanamycin or glucose as a single carbon source in the presence of DNJ (range of 0.00001–10 mM). Cultivability was measured by plating and counting CFUs during 24 h. We found that in the presence of DNJ and glucose, the bacteria showed initial growth retardation which was then rapidly overcome (**Figure 1A**). In contrast, adding DNJ to a minimal medium containing only kanamycin as a carbon source arrested growth completely. This suggested that glycosyl-hydrolases are required for the subsistence phenotype on kanamycin (**Figure 1B**).

### Kanamycin Degradation by *Escherichia coli*

Finally, we studied kanamycin degradation by *E. coli* (DH5α) in the presence or absence of the plasmid encoded APH (3) II gene using 1 mg/ml of high purity kanamycin (Evopure, 99.25%) in M9 medium. Bacterial growth was calculated using the plate counting method, and kanamycin was measured by LC-MS/MS. It was observed that the number of CFUs increased during the first 8 h, although no degradation of the antibiotic was observed (**Table 3**).

**Table 3:** Concentration of kanamycin and colony forming units (CFUs) obtained in M9 minimal media with kanamycin (EvoPure^TM^-1 mg/ml) with and without resistant *E. coli* during LC-MS/MS experiments over time.

**Samples**	**Kanamycin concentration (mg/L)**				**CFU (ml)**			
		
Time (Hours)	0	4	8	24	0	4	8	24
MM + KAN	978	1021	1066	1399	–	–	–	–
MM + *Ec* + KAN	923	977	1005	1306	6.6E + 07	5.4E + 07	5.4E + 07	4.0E + 07
MM + *Ec-p* + KAN	907	984	1008	1266	4.2E + 07	6.8E + 07	2.2E + 08	1.1E + 09


*MM, minimal media; KAN, Kanamycin; *Ec*, *Escherichia coli*; *Ec-p*, *Escherichia coli*-plasmid encoding aminoglycoside 3′ phosphotransferase II gene.*

## Discussion

We observed that two groups of bacteria, *E. coli* and *Cellulosimicrobium* sp., present in the gut microbiota of healthy human volunteers and zoo animals, displayed the subsistence phenotype on aminoglycosides and erythromycin as a single carbon source. The subsistence phenotype was defined as an increase of CFUs over multiple transfers compared to the inoculum incubated in the same media without a carbon source. In order to avoid the presence of residual carbon sources, we included a pre-washing step to prevent carry-over of dissolved carbon from the fecal material and used new sterile glass material and freshly prepared media. In addition, we included serial two-fold dilutions of glucose and kanamycin (1–0.0625 mg/ml) and observed the subsistence phenotype at all antibiotic concentrations including those more similar to amounts found in natural habitats (Trieu-Cuot and Courvalin, 1986; data not shown).

Subsistence phenotypes were found previously in *P. fluorescens* isolates obtained from lake sediments, which were described to utilize benzylpenicillin as a carbon, nitrogen and energy source (Johnsen, 1977). Soil bacteria from the orders *Pseudomonadales* and *Burkholderiales* have also been isolated based on their capacity to grow on a range of antibiotics as a single carbon source (Dantas et al., 2008). In another environment including clinical and non-clinical samples, Barnhill et al. (2011) observed that multi-resistant *Salmonella* spp. were also able to subsist on antibiotics, highlighting the potential prevalence of the antibiotic subsistence phenotype in a clinical context. Xin et al. (2012) showed that two members of the Enterobacteria group (e.g., *Klebsiella pneumoniae* and *Escherichia fergusonii*) isolated from fecal material of healthy volunteers were able to subsist and bio-degraded chloramphenicol as a sole carbon source. However, all the strains in the study were chloramphenicol susceptible, which indicates that the subsistence and resistance mechanisms were independent in this particular case.

In our study, since the majority of the bacteria seemed to subsist on aminoglycosides, we studied laboratory strains of *E. coli* and *P. putida* with a plasmid-encoded APH (3′) II gene in order to differentiate aminoglycoside resistance and the subsistence phenotype. Our results showed that a common resistance gene facilitates the subsistence phenotype on aminoglycosides, and these results indicated that resistance and subsistence mechanism might be linked. Similar subsistence phenotypes were obtained with *P. putida* TEC1 using the cloning vector pUTmini-Tn5-Km1 (de Lorenzo et al., 1990; Leprince et al., 2012), which similarly contains an APH (3′) II gene.

Previous studies have shown that kanamycin is stable under culture conditions for at least a week (Ryan et al., 1970). Stability has been attributed to its structure where a six-aminocyclitol ring is attached to aminosugar side chains through glycosidic bonds. We hypothesized that an intrinsic metabolic capacity to break down and utilize phosphorylated aminoglycosides is present in various bacteria. In the genomes of *E. coli* and *P. putida* a multitude of genes predicted to encode GH exist (40–50 in *E. coli* and 26 in *P. putida*), with typically between 20 and 22 GH gene families annotated in *E. coli*. The encoded enzymes could potentially be involved in breaking the glycosidic bonds in the aminoglycosides, releasing an accessible carbon source. Due to the large number of GH encoding genes though single and combinatorial gene knockouts would not be numerically feasible. It is also likely that this approach may not deliver the necessary result due to potential functional redundancy of these enzymes. In our study we showed that a specific glycosyl-hydrolase inhibiting iminosugar (DNJ) abolishes the subsistence phenotype on aminoglycosides. This suggests that glycosyl-hydrolase activity could be necessary for the hydrolysis of the glycosidic bond and subsequent release of the aminosugars from the aminoglycoside, and hence indicates an involvement of GH in the antibiotic subsistence phenotype.

Since we found several indications of aminoglycoside subsistence phenotypes in line with previous observations, we applied the LC-MS/MS method to study kanamycin degradation. However, no degradation of kanamycin was observed in our study. Our findings thus align with the previous observations by Walsh et al. (2013) suggesting that due to the lack of antibiotic degradation, the subsistence phenotype cannot be linked to the use of the antibiotic as a sole carbon source.

So far, no genes have been identified in the catabolic pathways of Kanamycin (http://www.ebi.ac.uk/chebi/chebiOntology.do?chebiId=CHEBI:6104). However, Stancu and Grifoll (2011), showed that several groups of Gram-positive and Gram-negative bacteria (including members of the *Enterobacteriaceae* family), displayed particular metabolic capabilities such as hydrocarbon degradation since these were able to grow on Poeni crude oil as a single carbon source. In addition, they show that Gram-negative bacteria possessed between two and four catabolic genes involved in degradation of saturated, monoaromatic, and polyaromatic hydrocarbons. Interestingly, these groups of bacteria were resistant to hydrophilic antibiotics such as ampicillin and kanamycin, and cellular and molecular modifications were induced by the antibiotic.

Since subsistence phenotypes on a range of antibiotics are readily observed, it is possible that antibiotic resistance genes frequently allow not only resistance, but also simultaneously facilitate antibiotic subsistence. Dantas and Sommer (2012) investigated the connection between subsistomes and resistomes, and indicated that thus far not a single gene involved in antibiotic subsistence has been identified. Although active aminoglycoside efflux pumps have been observed in *E. coli* (Mingeot-Leclercq et al., 1999), it is hypothesized that this mechanism is not actively involved in the *E. coli* clones subsisting on the antibiotics. This is because such activity would hinder accumulation of the drugs in the cytoplasm, where they are required for catabolism to occur.

Another potential subsistence mechanism that we considered was ribosomal protein mutations in spontaneous kanamycin resistant *E. coli* strains. It has been indicated that resistance to kanamycin and neomycin by ribosomal protein mutation is uncommon since this antibiotic binds to multiple sites on 30S and 50S ribosomal subunits, and high level resistance cannot be achieved by a single mutation (Kucers et al., 1997). However, aminoglycoside modifying enzymes encoded by plasmids including the acetyltransferases, adenyltransferases, and phosphotransferases encoded by plasmids (Neu, 1992) may inactivate antibiotics (i.e., by acetylation of amino groups, adenylation and phosphorylation of hydroxyl groups), before the subsequent action of the catabolic enzymes.

Based on our results we conclude that gut bacteria isolated were not able to degrade kanamycin and utilize it as a carbon source. Nevertheless, we observed that the presence of an aminoglycoside resistance gene supports the aminoglycoside subsistence phenotype, and GH seem to be required. This could indicate a possible link between the resistance and the subsistence phenotype. In addition, as we only tested one type of aminoglycoside modifying enzyme, we cannot assume that all the aminoglycoside modifying enzymes act in the same way. The different mechanisms of enzymatic modification could have different consequences. Further studies of kanamycin degradation linked to the evaluation of the subsistence phenotype and other aminoglycoside modifying enzymes may therefore provide further insight to the underlying subsistence mechanism.

Bacteria need to adapt to the growth medium in order to be able to metabolize the nutrients, and during the lag phase they are not completely inactive. They grow in size and develop primary metabolites (such as proteins, enzymes, and RNA) as well as coenzymes and division factors required for making new cells. These factors together with the mechanisms involved in antibiotic resistance could also be hypothesized to facilitate the antibiotic subsistence phenotype. It also may well be that bacteria simply need to be resistant to the antimicrobial in order to be able to exploit trace levels of non-toxic breakdown products. Future analyses including experimental evolution of antibiotic subsistence will help to further unravel the possible mechanisms involved in this phenotype. Nevertheless, since we were able to identify a bacterial strain that displayed the subsisting phenotype with both aminoglycoside (kanamycin) and macrolide (erythromycin) antibiotics, expansion of future studies to include resistance genes and metabolic pathways of macrolides as well as aminoglycosides could be of special interest.

## Author Contributions

TB: designed and performed the experiments, analyzed, and interpreted the data and wrote the paper. TZ and GB: performed the LC-MS/MS experiments, analyzed the data, and revised the work critically for intellectual content. HS and MP: supervised the project, substantial contribution to revising it critically, and final approval of the version to be published.

## Conflict of Interest Statement

The authors declare that the research was conducted in the absence of any commercial or financial relationships that could be construed as a potential conflict of interest.
